# The effect of CTLA-4 A49G polymorphism on rheumatoid arthritis risk: a meta-analysis

**DOI:** 10.1186/s13000-014-0157-0

**Published:** 2014-08-16

**Authors:** Gang Li, Fengjun Shi, Jingchen Liu, Ye Li

**Affiliations:** Department of Orthopaedics, Daqing General Hospital Group Oilfield General Hospital, Daqing, 163001 China; Department of Spine Surgery China-Japan Union Hospital, Jilin University, Changchun, 130031 China

**Keywords:** Rheumatoid arthritis, CTLA-4, Polymorphism

## Abstract

**Background:**

Recently, a number of studies have been performed to explore the association between CTLA-4 A49G polymorphism and rheumatoid arthritis (RA). However, the results of previous works are still controversial and ambiguous.

**Methods:**

In this work, we attempted to perform an updated meta-analysis of available case–control study in order to assess the association between CTLA-4 A49G polymorphism and RA risk. We searched the various citation databases without limits on languages. Article searching was performed by screening the references of retrieved studies manually. Odds ratios (OR) and 95% confidence intervals (95% CI) were calculated to evaluate the strength of the association.

**Results:**

We totally compiled 27 studies in 24 articles (9805 RA patients and 10691 control subjects) into our meta-analysis work. We found significant association between CTL-A4 A49G polymorphism and RA risk (GG *vs.* AA: OR = 1.13, 95% CI = 1.03–1.23; GA *vs.* AA: OR = 1.19, 95% CI = 1.07–1.33; GA + GG *vs.* AA: OR = 1.18, 95% CI = 1.07–1.29). In the subgroup analysis by ethnicity, evidences of significantly increased risk was also found in both Asian (GG *vs.* AA: OR = 1.34, 95% CI = 1.15–1.55; GA + GG *vs.* AA: OR = 1.24, 95% CI = 1.08–1.41) and Caucasian population (GA *vs.* AA: OR = 1.19, 95% CI = 1.03–1.37; GA + GG *vs.* AA: OR = 1.14, 95% CI = 1.01–1.29). No evidence of publication bias was found in this work.

**Conclusions:**

Our meta-analysis suggests that CTLA-4 A49G polymorphism was associated with RA risk.

**Virtual Slides:**

The virtual slide(s) for this article can be found here: http://www.diagnosticpathology.diagnomx.eu/vs/13000_2014_157

## Background

Rheumatoid arthritis (RA) is a common chronic inflammatory autoimmune disease characterized by significant disability and early mortality. RA affects ~1% of the adults worldwide [1]. Although its etiology has not been determined, RA has been regarded as a complex autoimmune disorder characterized by a chronic T-cell response. So, genes involved in T-cell response regulation might be important determinants of RA susceptibility.

The cytotoxic T-lymphocyte antigen 4 (CTLA-4) is an inhibitory receptor predominantly expressed on the activated and regulatory T lymphocytes [2]. It plays an important role in regulating T-cell activation. Several studies have documented that polymorphism of CTLA-4 A49G have remarkable effects on the susceptibility to autoimmunity [3]. A49G (rs231775) polymorphism, located in the first exon region of CTLA-4 gene, was identified as a functional single nucleotide polymorphism with a A to G change. This polymorphism has been shown to be associated with the susceptibility of diverse diseases [4-8], including RA. The results of previous reports on the possible association of CTLA-4 A49G polymorphism with RA risk remain controversial and ambiguous. To our knowledge, the issue of whether CTLA-4 A49G polymorphism can increase RA risk remains largely uncertain. A comprehensive meta-analysis can provide a reliable estimate in genetic association studies. In this work, we conducted a quantitative meta-analyses that increased statistical power to derive a more precise estimation of the relationship.

## Methods

### Study identification and selection

A systematic literature search was carried out in the PubMed, IS Web and Chinese National Knowledge Infrastructure (CNKI) database with the terms of “CTLA-4”, “rheumatoid arthritis”, “polymorphism”, “variation”. Two investigators independently screened the information including the titles, abstracts and full texts to determine inclusion carefully. No language restrictions were used in our literature search.

### Data extraction

The following inclusion criteria were used to select related literatures for the meta-analysis: (1) studies about the association between CTLA-4 A49G polymorphism and RA risk; (2) a case-controlled RA study of CTLA-4 A49G polymorphism with complete genotype distribution data; (3) studies on sufficient data and the original data from case–control studies. The following information were extracted from included studies: first author, year of publication, original country, ethnicity of the sample and genotype distributions.

### Statistical analysis

The pooled odds ratios (ORs) with the corresponding 95% confidence intervals (95% CI) were used to evaluate the strength of association between the polymorphism of CTLA-4 A49G and RA risk. Four models were estimated: the co-dominant model (GG *vs.* AA, GA *vs.* AA), dominant model (GA + GG *vs.* AA) and recessive model (GG *vs.* GA + AA), respectively. Subgroup analyses were performed by ethnicity.

At first, we evaluated the HWE in the controls for each study using chi-square and a P < 0.05 was considered as significant disequilibrium. Statistical heterogeneity among the studies was gauged by the Chi-square based on Q-test. A P value greater than 0.1 for the Q-test indicates no significant heterogeneity existing among studies, and the pooled OR estimation was performed using the fixed-effects model (the Mantel-Haenszel method). Otherwise, the random-effects model (DerSimonian and Laird method) was used. Potential publication bias of literatures was analyzed through the Egger’s linear regression test with a funnel plot. All statistical analyses were performed with STATA version 11.0 (Stata Corporation, College Station, TX). All the *P* values were calculated using a two-sided test and *P* < 0.05 were considered as statistically significant.

## Results

### Study characteristics

A total of xx articles were retrieve after the first search in various databases. After literature selection, 43 studies about the association of CTLA-4 A49G polymorphism with RA risk were identified and screened for data retrieval. Among which, 16 studies were irrelevant. At last, a total of 27 eligible independent case–control studies in 24 articles were included [9-33]. The characteristics of selected studies are summarized in Table 1. All of the studies indicated that the genotypic distribution of the controls was consistent with Hardy-Weinberg equilibrium.

**Table 1:** **Main characteristics of studies included in our work**

**First author**	**Year**	**Location**	**Ethnicity**	**Case/controls**			**HWE**
				**GG**	**GA**	**AA**	
Seidl	1998	Germany	Caucasian	37/68	138/210	83/179	0.88
Matsushita	1999	Japan	Asian	200/56	199/72	62/22	0.98
Gonzalez-Escribano	1999	Spain	Caucasian	10/30	63/103	65/172	0.06
Barton	2000	Spain	Caucasian	14/12	57/70	65/62	0.44
Yanagawa	2000	Japan	Asian	29/78	50/88	6/34	0.56
Hadj	2001	Tunisia	African	23/68	27/62	10/20	0.33
Milicic	2001	UK	Caucasian	63/73	223/213	135/166	0.94
Lee	2002	Korea	Asian	41/49	35/29	10/8	0.49
Vaidya	2002	UK	Caucasian	20/45	65/158	38/146	0.97
Lee	2003	China	Asian	103/85	67/100	16/18	0.32
Liu	2004	Taiwan	Asian	14/21	42/50	9/10	0.07
Barton	2004	UK	Caucasian	34/29	55/68	43/59	0.49
Lei	2005	China	Asian	148/86	138/125	40/39	0.84
Takeuchi	2006	Japan	Asian	49/44	39/49	12/11	0.88
Suppiah	2006	UK	Caucasian	40/92	144/241	92/142	0.85
Tsukahara	2008	Japan	Asian	636/181	668/194	186/73	0.23
Walker	2009	Canada	Caucasian	177/179	554/576	409/493	0.87
Munoz-Valle	2010	Mexico	Mexican	42/34	102/82	55/83	0.22
Plant (F)	2010	France	Caucasian	96/15	315/75	273/72	0.77
Plant (Ge)	2010	Germany	Caucasian	37/94	111/83	72/83	0.44
Plant (Gr)	2010	Greece	Caucasian	26/33	133/107	113/147	0.15
Plant (U)	2010	UK	Caucasian	146/410	451/1255	407/994	0.91
Benhatchi	2011	Slovakia	Caucasian	6/5	33/25	18/21	0.82
Rocha	2011	Brail	Caucasian	9/3	29/15	32/12	0.86
Tang	2013	China	Asian	652/474	642/535	195/191	0.154
Alfadhli	2013	Kuwait	Asian	10/14	30/86	74/182	0.65
Liu	2013	China	Asian	77/130	111/125	25/48	0.16

### Quantitative synthesis

**Figure 1:**
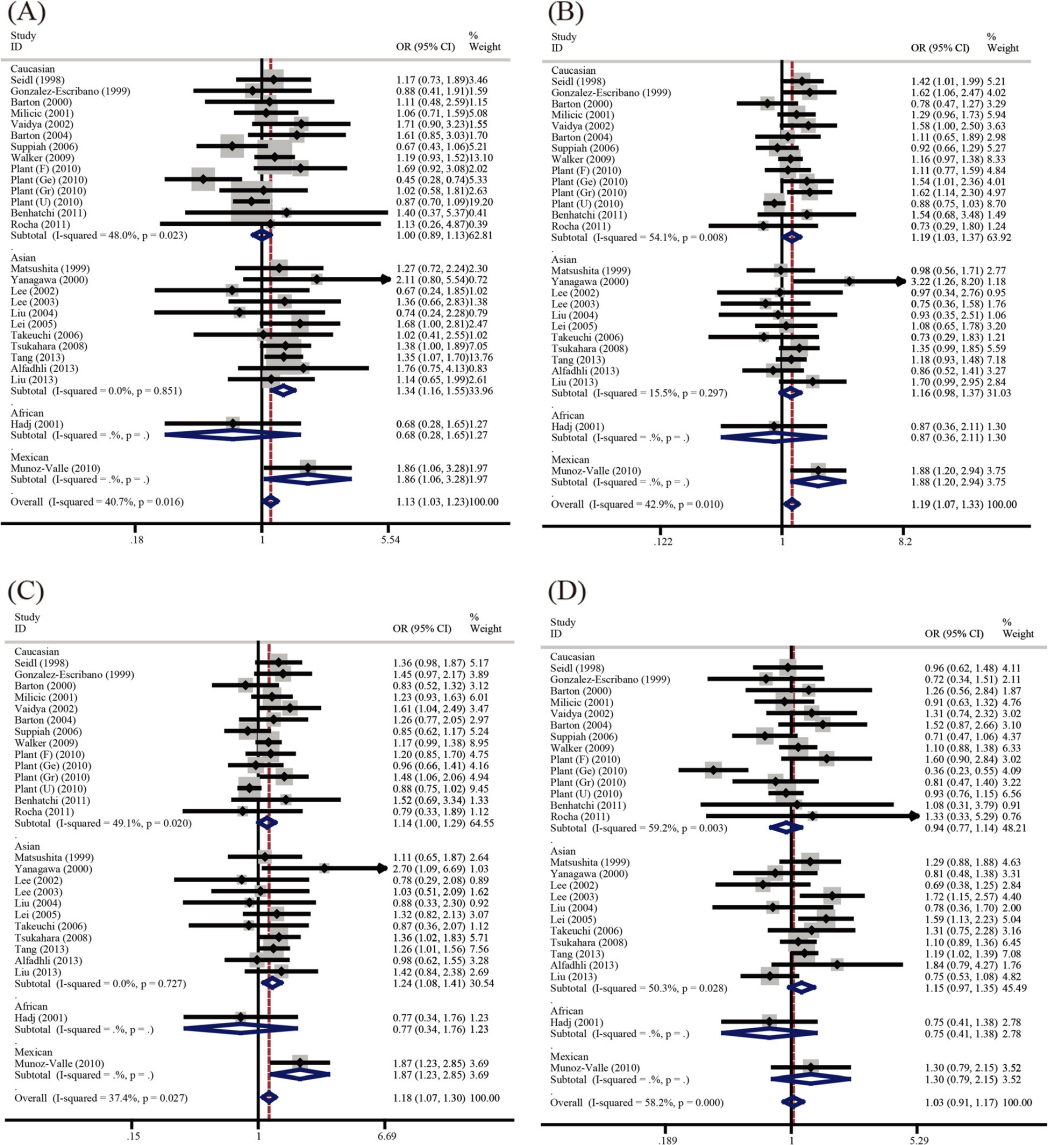
**Forest plots for the overall association between CTLA-4 A49G polymorphism and RA risk A) GG** ***vs.*** **AA; B) GA** ***vs.*** **AA; C) GA + GG** ***vs.*** **AA; D) GG** ***vs.*** **GA + GG.**

All of the main results of the meta-analysis were shown in Table 2. Overall, significant associations between CTLA-4 A49G polymorphism and RA risk was found (GG *vs.* AA: OR = 1.13, 95% CI = 1.03–1.23; GA *vs.* AA: OR = 1.19, 95% CI = 1.07–1.33; GA + GG *vs.* AA: OR = 1.18, 95% CI = 1.07–1.29) (Figure 1). Next, we performed a further analysis on data stratified by ethnicity groups with the attempt to search for possible factors that might impact the results. In the subgroup analysis by ethnicity, evidences of significantly increased risk was also found in both Asian (GG *vs.* AA: OR = 1.34, 95% CI = 1.15–1.55; GA + GG *vs.* AA: OR = 1.24, 95% CI = 1.08–1.41) and Caucasian population (GA *vs.* AA: OR = 1.19, 95% CI = 1.03–1.37; GA + GG *vs.* AA: OR = 1.14, 95% CI = 1.01–1.29) in different genetic models.

**Table 2:** **Results of meta-analysis for CTLA-4 A49G polymorphism and RA risk**

**Groups**	**GG vs AA**		**GA vs AA**		**GA + GG vs AA**		**GG vs GA + AA**	
	***P-value***	**OR (95% CI)**	***P-value***	**OR (95% CI)**	***P-value***	**OR (95% CI)**	***P-value***	**OR (95% CI)**
Total	0.009	1.13 (1.03, 1.23)	0.001	1.19 (1.07, 1.33)	0.001	1.18 (1.07, 1.29)	0.66	1.03 (0.91, 1.17)
Asians	0.001	1.34 (1.15, 1.55)	0.083	1.16 (0.98, 1.37)	0.002	1.24 (1.08, 1.41)	0.099	1.14 (0.97, 1.35)
Caucasian	0.991	1.00 (0.89, 1.12)	0.015	1.19 (1.03, 1.37)	0.042	1.14 (1.01, 1.29)	0.51	0.93 (0.77, 1.14)

### Heterogeneity analysis

Significant heterogeneity existed in all four genetic models (GG *vs.* AA: *P* = 0.016, I^*2*^ = 40.7%; GA *vs.* AA: *P* = 0.01, I^*2*^ = 42.9%; GA + GG *vs.* AA: *P* = 0.027, I^*2*^ = 37.4%; GG *vs.* GA + AA: *P* = 0.00, I^*2*^ = 58.2%). Therefore, the random-effect models were employed in all genetic models.

### Sensitivity analyses and publication bias

**Figure 2:**
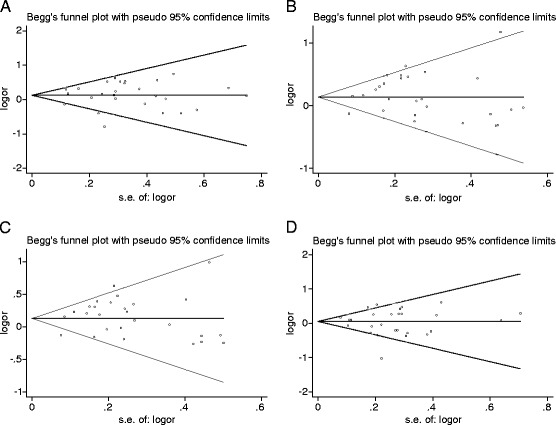
**Funnel plots for A49G polymorphism of CTLA-4 in RA disease A) GG** ***vs.*** **AA; B) GA** ***vs.*** **AA; C) GA + GG** ***vs.*** **AA; D) GG** ***vs.*** **GA + GG.**

Sensitivity analyses were performed to assess whether each individual study can affect the final results by using Begg’s test and Egger’s test. Neither the Begg’s test nor the Egger’s test provided any obvious evidences of publication bias (Table 3). The shapes of the funnel plots appeared to be symmetrical in all genetic models (Figure 2). These results showed that no individual study affected the final results in diverse genetic models using the exclusion method step by step.

**Table 3:** **Results of Egger’s test and Begg’s test**

**Comparison**	**Egger’s test**			**Begg’s test**	
	***t***	***P***	**95% CI**	***Z***	***P***
GG vs AA	0.31	0.76	(−0.92, 1.25)	0.67	0.51
GA vs AA	1.02	0.32	(−0.54, 1.61)	0.33	0.74
GA + GG vs AA	1.07	0.29	(−0.49, 1.55)	0.67	0.51
GG vs GA + AA	−0.55	0.58	(−1.62, 0.94)	0.08	0.93

## Discussion

Although many efforts have been devoted for decades, the underlying molecular genetic basis of RA remains largely unknown. Recently, researches in the genetic susceptibility to RA have led to growing attentions to the study of association between gene polymorphisms and RA [34]. Large and well-designed genotype-phenotype investigations with robust statistical technical are required to detect these mild to moderate associations. Up to date, the association studies have been performed for CTLA-4 A49G polymorphism with respect to several disease susceptibility, such as liver disease, pancreatic cancer, primary biliary cirrhosis, etc. [5,35,36]. It has been realized that CTLA-4 A49G polymorphism play important role in disease disorders.

Several case–control studies have demonstrated inconsistent and even inverse relationship between CTLA-4 A49G polymorphism and RA risk. Until now, there are still no consensus results on the association of CTLA-4 A49G polymorphism and susceptibility of RA. In this work, we conducted a more comprehensive meta-analysis on the CTLA-4 A49G polymorphism and RA. For the overall data, it has been shown that CTLA-4 A49G polymorphism have an elevated association with RA risk. In the subgroup analysis by ethnicity, results suggested that strong evidences support the association between RA risk and CTLA-4 A49G polymorphism in both Asian and Caucasian populations. Previous meta-analyses works have reported that the CTLA-4 A49G polymorphism is associated with RA risk in Asians, but not in Caucasians using limited data [37,38]. In our work, we performed an up-dated meta-analyses, and found that A49G polymorphism is associated with RA risk in both Asians and Caucasians.

Many works have been devoted to examine the association between polymorphisms and RA risk [39,40]. As we know, genetic polymorphism of biomarkers is the key factor leading to the susceptibility of diseases. Our work suggests that CTLA-4 A49G polymorphism might be a potential clinical marker for RA. Important clinical insights are emerging, and this polymorphism provides new understanding of RA diagnostic advances. We provided useful reference for clinical medical treatment. However, there are still some limitations in our meta-analysis work. First, we were not able to take into account other factors because of lacking the original data, such as alcohol addictive, inflammation and other disease that may influence the association estimates; Second, although all eligible studies were summarized, the total sample size might have not been enough to make a convincing conclusion. So, when we performed stratified analysis of ethnicity, the number of each subgroup was relative smaller.

## Conclusions

Our meta-analyses provided a more comprehensive evidence of the association between CTLA-4 A49G polymorphism and RA risk. The result showed that CTLA-4 A49G polymorphism is associated with susceptibility of RA.
